# Impact of Regorafenib on Endothelial Transdifferentiation of Glioblastoma Stem-like Cells

**DOI:** 10.3390/cancers14061551

**Published:** 2022-03-18

**Authors:** Pauline Deshors, Florent Arnauduc, Betty Boëlle, Elizabeth Cohen-Jonathan Moyal, Monique Courtade-Saïdi, Solène M. Evrard

**Affiliations:** 1 Institut Claudius Regaud, IUCT Oncopole, 31059 Toulouse, France; pauline.deshors@inserm.fr (P.D.); bboelle@gmail.com (B.B.); moyal.elizabeth@iuct-oncopole.fr (E.C.-J.M.); 2 Faculty of Medicine, Paul Sabatier University, Toulouse-3, 31062 Toulouse, France; florent.arnauduc@univ-tlse3.fr (F.A.); monique.courtade-saidi@univ-tlse3.fr (M.C.-S.); 3 INSERM UMR 1037, Centre for Cancer Research of Toulouse, 31100 Toulouse, France; 4 Pathology and Cytology Department, CHU Toulouse, IUCT Oncopole, 31059 Toulouse, France

**Keywords:** regorafenib, glioblastoma, glioblastoma stem-like cells, transdifferentiation, tumour-derived endothelial cells, ionising radiation

## Abstract

**Simple Summary:**

Glioblastomas (GBM) are the most frequent and aggressive adult brain tumours with a poor prognosis despite heavy therapy that combines surgical resection and radio-chemotherapy. There is a real therapeutic failure concerning these tumours and it highlights the need for new efficient and usable molecules in these particular and not easily reachable cancers. GBM are also characterised by abnormal and abundant vascularisation, of which some vessels originate from transdifferentiation of GBM stem cells (GSC) to tumour-derived endothelial cells (TDEC). As this mechanism was described as essential for tumour growth, we evaluated the impact of regorafenib, a multikinase inhibitor with anti-angiogenic and anti-tumourigenic activity on transdifferentiation but also directly on GSC. Regorafenib significantly decreases GSC proliferation in vitro but also in vivo. Regorafenib also inhibits transdifferentiation by decreasing phenotypic and functional endothelial features of TDEC obtained from non-irradiated GSC but also of TDEC obtained from irradiated GSC. All these results confirm that regorafenib clearly impacts GSC tumour formation and transdifferentiation and may therefore be a promising therapeutic option in combination with chemo/radiotherapy for the treatment of these highly aggressive brain tumours.

**Abstract:**

Glioblastomas (GBM) are aggressive brain tumours with a poor prognosis despite heavy therapy that combines surgical resection and radio-chemotherapy. The presence of a subpopulation of GBM stem cells (GSC) contributes to tumour aggressiveness, resistance and recurrence. Moreover, GBM are characterised by abnormal, abundant vascularisation. Previous studies have shown that GSC are directly involved in new vessel formation via their transdifferentiation into tumour-derived endothelial cells (TDEC) and that irradiation (IR) potentiates the pro-angiogenic capacity of TDEC via the Tie2 signalling pathway. We therefore investigated the impact of regorafenib, a multikinase inhibitor with anti-angiogenic and anti-tumourigenic activity, on GSC and TDEC obtained from irradiated GSC (TDEC IR+) or non-irradiated GSC (TDEC). Regorafenib significantly decreases GSC neurosphere formation in vitro and inhibits tumour formation in the orthotopic xenograft model. Regorafenib also inhibits transdifferentiation by decreasing CD31 expression, CD31+ cell count, pseudotube formation in vitro and the formation of functional blood vessels in vivo of TDEC and TDEC IR+. All of these results confirm that regorafenib clearly impacts GSC tumour formation and transdifferentiation and may therefore be a promising therapeutic option in combination with chemo/radiotherapy for the treatment of highly aggressive brain tumours.

## 1. Introduction

Primary tumours of the central nervous system represent approximately 2 to 3% of all human cancers. Multi-form glioblastoma (GBM), classified as a grade IV astrocytoma according to the classification of the World Health Organisation, is the most aggressive and most common of these tumours in adults [1]. In fact, despite aggressive treatment with chemotherapy and radiotherapy, the average survival still remains between 12 and 15 months [2,3]. One of the main clinical characteristics of these tumours is their recurrence even within irradiation fields. A better understanding of the molecular and cellular mechanisms related to the resistance and recurrence of GBM is therefore a major stake. Thus, the presence of chemo-resistant, radio-resistant tumour cells appears to be responsible for these aggressive recurrences. Indeed, it is now established that GBM are highly heterogeneous tumours similar to most solid cancers [4]. Studies have highlighted the presence of a subpopulation of self-renewing and pluripotent GBM stem-like cells (GSC), also called GBM-initiating cells, in the tumour [5]. These GSC are characterised by their ability to self-renew in vitro (neurosphere formation) and in vivo, their expression of neural stem cell markers and stem cell transcription factors, their pluripotent aptitude to differentiate into brain cells and their high tumourigenic potential in vivo in orthotopical xenograft mice [6,7]. In addition, the presence of these GSC may explain the high GBM recurrence rate, since they have been shown to be extremely tumourigenic and radio resistant.

GBM is also characterised by significant vascularisation. The formation of tumour-related vessels develops prematurely during tumour progression. Although abundant, this vascularisation is morphologically and functionally abnormal. Abnormal vascularisation potentiates the onset of hypoxia within the tumour, thereby assisting in stem-related GSC maintenance [8]. Various mechanisms of glioma-associated neovascularisation have been described, such as vascular co-option, angiogenesis, vasculogenesis, vascular mimicry and transdifferentiation [9]. The endothelial transdifferentiation of GBM stem-like cells was described by different teams and proved to be essential for tumour growth [10,11,12]. Moreover, De Pascalis et al. demonstrated more recently that this mechanism is enhanced after irradiation [13]. We confirmed these results and showed that the Tie2 signalling pathway is involved in this irradiation-induced transdifferentiation [14].

Regorafenib is an oral tyrosine kinase inhibitor that blocks multiple protein kinase activities, including proteins involved in the regulation of oncogenesis (KIT, RET, RAF-1 and BRAF), tumour microenvironment (PDGFR and FGFR) and tumour angiogenesis (VEGFR1, 2 and 3 and TIE-2) [15,16]. Regorafenib is currently approved for the treatment of refractory metastatic colorectal cancer, advanced gastrointestinal tumours (GIST) previously treated with imatinib and sunitinib and unresectable hepatocellular carcinoma following sorafenib progression [17]. Several recent preclinical studies and clinical trials have shown that regorafenib is active against other tumour types including gastric cancer, sarcomas (other than GIST), biliary tract cancers and brain tumours [17]. In fact, Daudigeos-Dubus et al. showed that regorafenib significantly inhibited tumour growth in all nervous system-derived xenografts such as medulloblastoma, neuroblastoma, high-grade glioma and glioblastoma [18]. They showed that antitumour activity was partly linked to decreased tumour vascularisation. Indeed, other studies have demonstrated the important inhibition of regorafenib on angiogenesis using normal endothelial cells such as HUVEC or HULEC or other tumour xenograft models [15,16]. Indeed, regorafenib has displayed anti-angiogenic effects in a human colorectal xenograft model as well as in a ratGS9L glioblastoma model [15]. Moreover, the efficacy and safety of regorafenib in the treatment of recurrent glioblastoma were assessed in a comparative, randomised phase II trial (REGOMA). This study shows that regorafenib significantly increases overall survival in patients with glioblastoma after radiotherapy and temozolomide have failed [19].

The encouraging clinical benefits of regorafenib monotherapy associated with our previous work, showing the cardinal role of the Tie2 signalling pathway in irradiation-induced transdifferentiation, led us to study the impact of this pan-tyrosine kinase inhibitor on endothelial transdifferentiation of GSC both in vitro and in vivo.

## 2. Materials and Methods

### 2.1. Human Tumour Collection

The study was conducted on newly diagnosed GBM tumour samples isolated from 2 different patients to establish 2 primary GSC cell lines (GC1 and GC2). All samples were obtained as part of the STEMRI clinical trial (NCT01872221) with written informed consent from patients admitted to the Neurosurgery Department at Toulouse University Hospital and were processed in accordance with the establishment’s Human Research Ethics Committee. The tumours used in this study were histologically diagnosed as grade IV astrocytoma according to the WHO criteria. GC1 were isolated from a proneural subtype tumour and GC2 from a classical subtype tumour.

### 2.2. Cell Culture and Treatments

The GBM samples were processed as described previously [14,20,21,22,23] in order to obtain GSC-enriched primary neurospheres. Neurosphere GSC were maintained in stem cell medium (SCM) at 37 °C in 5% CO_2_. The stem cell medium comprised DMEM-F12 (Lonza, Bâle, Swiss) supplemented with B27 and N2 (Invitrogen/ThermoFischer Scientific, Waltham, MA, USA), 25 ng/mL of FGF-2 and EGF (Peprotech, Neuilly-sur-Seine, France). 

HUVEC (Human Umbilical Vein Endothelial Cells, Lonza) were cultured in EGM-2 medium (Endothelial Growth Medium, Lonza). 

GSC neurospheres were dissociated for transdifferentiation. Cells were then cultured and plated as an adherent monolayer (at least 4 × 10^3^ cells/cm^2^) in EGM-2 medium (transdifferentiation medium) for 15 days to obtain TDEC (tumour-derived endothelial cells). The HUVEC and TDEC were collected after trypsinisation for subsequent experiments.

### 2.3. Preparation of Regorafenib

Regorafenib (BAY 73–4506; Bayer Pharma AG, Berlin, Germany) was stored as a solid substance. For in vitro experiments, regorafenib was dissolved in 100% dimethyl sulfoxide (DMSO) and diluted in the complete medium. For in vivo experiments, regorafenib was formulated as a solution in polypropylene glycol/PEG400/Kolliphor 188 (42.5/42.5/15 plus 20% distilled water). All compound preparations were stored at room temperature in the dark. Solutions were freshly prepared every week.

### 2.4. Orthotopic Xenograft Generation

Nude mice were housed in the regional center for functional exploration and experimental resources facility. The approval of the Institute’s Animal Ethics Committee was obtained for the use of the animal model and the study protocols. Nude mice were used as per protocol (APAFlS#7660-2016110818123504 v2), duly reviewed and approved by the Institutional Animal Care and Use Committee of the Midi-Pyrenees region (France). 

Orthotopic human GBM xenografts were established in 4–6-week-old female nude mice (Janvier Labs, Le Genest-Saint-Isle, France) as previously described [20,21]. Briefly, mice received a stereotactically guided injection of 2.5 × 10^5^ GC1 resuspended in 5 µL of DMEM-F12. The injection was accurately delivered into the right forebrain (2 mm lateral and 1 mm anterior to the bregma, at a depth of 5 mm from the skull surface). One month after surgery, regorafenib was administered orally by gavage at the dose of 30 mg/kg per day for 45 days. The control animals received the vehicle. The mice were sacrificed 10 days after the last dose and excised brains were collected, fixed, cut horizontally and included in the same block of paraffin. The sections were deparaffinised in xylene and then rehydrated in an ethanol series for histological analysis. Staining with Mayer’s haematoxylin (Merck) and eosin-orange G (Merck) (HE) was performed prior to dehydration and mounting with Eukitt solution and examination on a Nikon ECLIPSE TS100. Serial sections were produced and 1 in every ten was stained with HE. The area with the main tumour volume was selected for immunohistochemistry.

### 2.5. Immunohistochemistry

IHC was performed on paraffin-embedded sections (5 µm) of excised brains of xenograft mice. The sections were incubated at 57 °C for 20 min followed by rehydration in xylene and ethanol series. Antigen retrieval was performed at 95 °C for 20 min using DAKO EnVision buffer pH9 (Agilent Technologies, Santa Clara, CA, USA) and washed with Wash Buffer 1X En Vision (Agilent Technologies). The slides were blocked with peroxidase blocking reagent En Vision (Agilent Technologies; 5 min), washed with PBS, incubated for 30 min with primary antibodies diluted in antibody diluent En Vision (Agilent Technologies) and then washed with Wash Buffer 1X En Vision (Agilent Technologies). The primary antibodies used were mouse anti-nestin (Novus Biologicals, Centennial, CO, USA; NB300-266) and rabbit anti-CD31 (Abcam; ab182981, Cambridge, UK). Slides were then incubated with FLEX/HRP (EnVision; 20 min), washed (Wash Buffer 1X (EnVision)) and revealed with DAB (diaminobenzidine peroxidase buffer) mixed with FLEX/DAB chromogen (EnVision; 5 min). Sections were then washed, counterstained with Mayer’s haematoxylin (Merck, Darmstadt, Germany), dehydrated and mounted with Eukitt solution before being viewed on a Nikon microscope. The size of each tumour was determined by measuring the area (mm^2^) of nestin-positive tumour cells on both sides of the brain using NDP.view software (Hamamatsu). The numbers of functional CD31+ vessels were counted in at least 3 random fields in the nestin-positive tumour area of each mouse under 63× magnification. The results are presented as the average number of CD31+ vessels in each condition (vehicle or regorafenib; 4 mice/group).

### 2.6. Neurosphere Formation Analysis

We used a clonogenic neurosphere-counting technique to assess the effect of regorafenib on GSC and neurosphere formation under treatment. After dissociation of the neurospheres, 250 cells/well were seeded in a 96-well plate and exposed to different doses of regorafenib (0, 1, 2, 3, 5 µM) in stem cell medium. The plates were incubated at 37 °C in 5% CO_2_. The number of neurospheres was counted after 7 days.

### 2.7. Cell Proliferation Analysis

Dissociated GSC were plated in 25 cm^2^ flasks at a density of 1 × 10^5^ cells per flask in EGM-2 medium containing regorafenib or DMSO, as the control, at a final concentration of 1 µM or 2 µM. The cells were then incubated at 37 °C in 5% CO_2_. After 6 days, the cells were harvested by trypsinisation, and the total number of live cells per condition was determined using an automated cell counter (Countess II FL, Life Technologies, Carlsbad, CA, USA).

### 2.8. Western Blotting

Cells were lysed in RIPA buffer complemented with protease and phosphatase inhibitor cocktails (Sigma-Aldrich, Saint Louis, MO, USA). The protein content was quantified using Bradford Reagent (BioRad, Hercules, CA, USA) and 30 to 50 µg of protein were then applied to a 7.5% or 10% SDS-PAGE, electroblotted onto PVDF membranes (Amersham Biosciences, Amersham, UK). The membranes were then blocked with 10% milk for 1 h. The primary antibodies used were mouse anti-GAPDH (Calbiochem, Sigma-Aldrich, Saint Louis, MO, USA; cat#CB1001, 1/10,000), mouse anti-CD31 (Abcam, Cambridge, UK; ab9498, 1/1000), mouse anti-Tie2 (Santa-Cruz Biotechnology, Dallas, TX, USA; sc-293414, 1/200), mouse anti-phosphoTie2 (phospho Y992) (Abcam, Cambridge, UK; ab192800, 1/1000), rabbit anti-AKT (Cell Signaling, Danvers, MA, USA; cat#4691, 1/2000) and Rabbit anti-phospho AKT (Ser473) (Cell Signaling, Danvers, MA, USA; cat#4060, 1/1000). Primary antibodies were incubated overnight, and the membranes were then washed. After incubation with HRP-linked secondary antibodies (anti-mouse (Abcam, 1/10,000), anti-rabbit (Abcam, 1/10,000)), the reaction was developed with Western ECL substrate (ThermoFischer Scientific, Waltham, MA, USA). The bands were revealed using ChemiDoc Imaging System (BioRad) and quantifications were realised using Image Lab^TM^ software (BioRad, Hercules, CA, USA) or ImageJ software. Original uncropped immunoblots from all Figures in this manuscript are presented as Supplementary Figures S8–S13.

### 2.9. CD31 Cell Expression Quantification by Flow Cytometry Analysis

After 15 days of transdifferentiation, samples of 2 × 10^5^ cells were incubated for 30 min in PBS with 10% FCS at 4 °C to avoid non-specific binding and then incubated with anti-human CD31-APC monoclonal antibody (Invitrogen 17-0319, 1/60) for 1 h at 4 °C in the dark. Fluorescent immunolabelling was measured using a MacsQuant10 (Miltenyi Biotec) flow cytometer and at least 10,000 events were recorded for each sample. 

The antibody Mouse IgG1 K Isotype Control APC (Invitrogen, 17-4714, 1/60) served as the control.

### 2.10. Pseudotube Formation Assay 

Pseudotube formation and quantification were performed by dropping 80 µL of Growth Factor Reduced Matrigel ™ (Corning) for polymerisation into the wells of a 96-well plate for 1 h at 37 °C. The cells were added at 1.5 × 10^4^ cells per well to the growth medium and incubated for 24 to 48 h at 37 °C in 5% CO_2_. To quantify the pseudotube formation, at least 3 fields were photographed (Nikon NIS Element camera, 10× magnification), and the overall pseudotube length per field was determined by measuring each tube using ImageJ software. 

### 2.11. Irradiation

Dissociated GSC were maintained in stem cell medium for 6 h and subjected to a 2 Gy irradiation dose, equivalent to the daily dose used for GBM patients, with an irradiator (XRAD Smart Plus (PXI)). After irradiation, GSC were kept in stem cell medium for 24 h to recover. The cells were then placed in transdifferentiation medium for 15 days.

### 2.12. In Vivo Matrigel™ Plug Assay

Nude mice were used in accordance with protocol APAFIS#10654-2017071812393169 v2, reviewed and approved by the Institutional Animal Care and Use Committee of the Midi-Pyrénées Region (France). 

After 15 days of culture in transdifferentiation medium, 5 × 10^6^ cells were mixed on ice with Growth Factor Reduced Matrigel™ (Corning/Sigma-Aldrich, Saint Quentin Fallavier, France). This mixture was then injected subcutaneously into nude mice in the dorsolateral region. 

Regorafenib was administered orally by gavage at 30 mg/kg daily; the controls received the vehicle. After 14 days, the mice were sacrificed and the plugs were collected, fixed and embedded in paraffin. The sections were deparaffinised in xylene for histological analysis and then rehydrated in the ethanol series. Staining with Masson’s trichrome was performed prior to dehydration followed by mounting with Eukitt solution. The sections were examined beneath a Nikon ECLIPSE TS100. The number of functional blood vessels was determined by counting the number of blood vessels containing red blood cells on photographs of at least 5 random fields per plug. 

### 2.13. Statistical Analysis

For the cell culture, each individual experiment was repeated at least three times. Where appropriate, mice were randomised into experimental groups by random number generator. Statistical power was determined from prior laboratory studies using similar models/cells [14,24]. Data were normalised, as appropriate, to mean values of each experiment with the reference condition set at 1. Experimental data were analysed using the unpaired two-tailed *t*-test. The results were expressed as the mean ± SEM. Differences were deemed significant at *p* < 0.05. These analyses were performed using Prism, Version 5.00 (GraphPad Software) or STAT VIEW software package (SAS, Cary, NC, USA).

## 3. Results

### 3.1. Regorafenib Inhibits the Tumourigenic Potential of GSC

**Figure 1:**
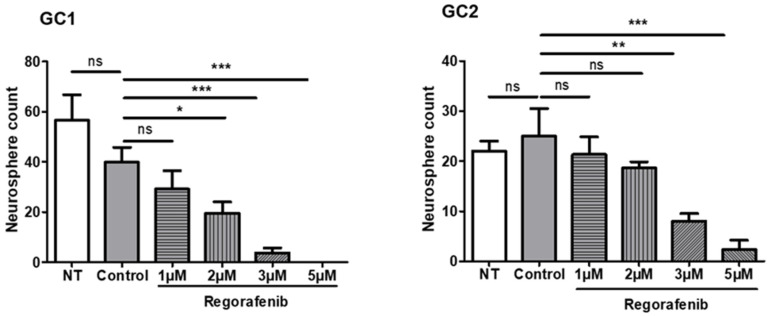
Regorafenib inhibits neurosphere formation in vitro. After dissociation, 250 cells/well, each GSC line was placed in 96-well plates and exposed to different doses of regorafenib (0 (vehicle only known as the control), 1, 2, 3, 5 µM) or without regorafenib (NT: not treated) in stem cell medium. The number of neurospheres was counted in each well after incubating for 7 days at 37 °C. The results are expressed as the mean ± SEM of at least three independent experiments. ns: not significant; * *p* < 0.05; ** *p* < 0.01; *** *p* < 0.001.

The self-renewal ability, a major stem characteristic, was assessed by assessing the neurosphere-forming capacity of GSC at low cell density in the presence of regorafenib. For that purpose, we used two different primary GSC (GC1 and GC2) established from surgical GBM samples collected from two patients and fully characterised in our previous work [20,21,22,23]. We treated GSC with different doses of regorafenib (0, 1, 2, 3, 5 µM) and counted the number of neurospheres seven days later. As shown in Figure 1, we observed a dose-dependent decrease in neurosphere formation in the two different GSC tested. This decrease was significant with 2 µM of regorafenib in GC1 and with 3 µM in GC2. However, in both GSC, 3 µM triggered a 50% decrease in neurosphere formation. 

**Figure 2:**
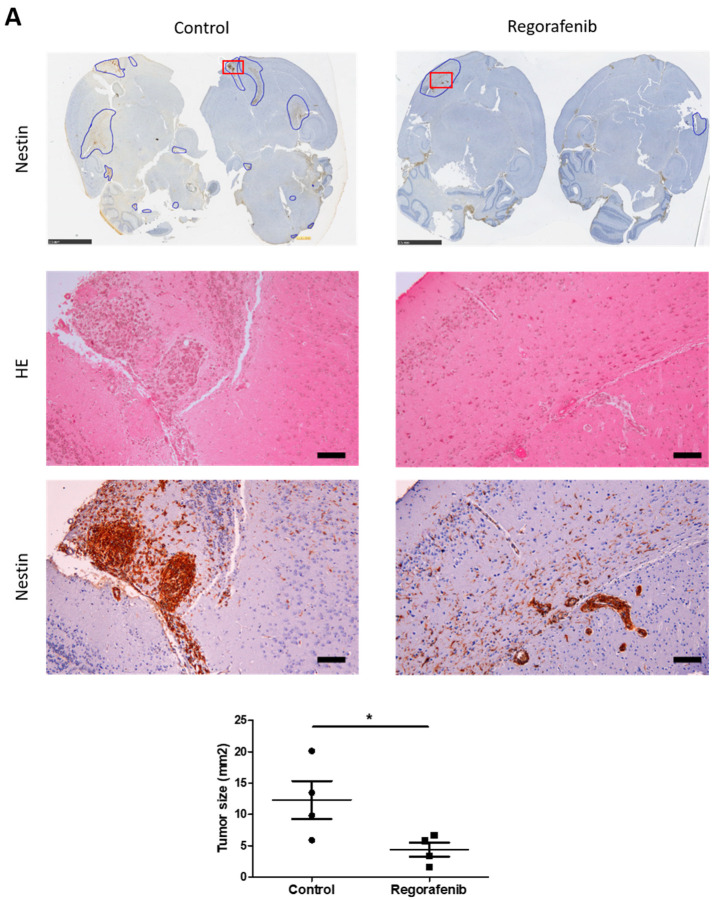

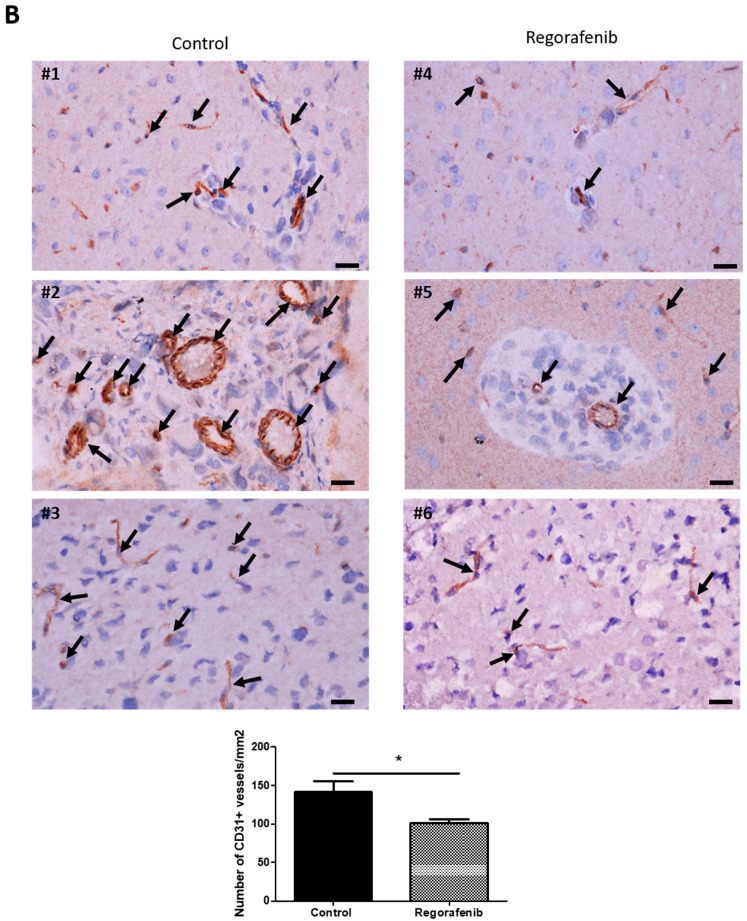
Regorafenib inhibits tumour growth and vascularisation in xenograft mice. GC1 were implanted into the right forebrain of mice. The mice then received daily oral vehicle or regorafenib at 30 mg/kg for 45 days. (**A**) Representative photographs of mice brain tumours treated with regorafenib (right panel) or without regorafenib (left panel) stained by hemalun–eosin (median panel) or with nestin antibody (upper and lower panel). The upper panel shows whole brains of mice stained with nestin antibody. The tumour areas were circled in blue, and each surface was measured in mm^2^. The red rectangles indicate the zone of the tumour shown in median (hemalun–eosin staining) and lower (nestin IHC) panel. Scale bars, upper panel 2.5 mm, median and lower panel 100 µm. The graph shows the tumour size in mm^2^ (expressed as the mean ± SEM of 4 mice). (**B**) Representative immunohistochemistry photographs of CD31+ vessels in brain tumour areas of 3 different mice treated with regorafenib (right panel) or without regorafenib (control, left panel). Arrows indicate CD31+ blood vessels. Scale bars, 20 µm. The graph shows the number of CD31+ vessels in tumours per mm^2^ (expressed as mean ± SEM of 4 mice). * *p* < 0.05.

Another important characteristic of GSC is their high tumourigenicity, especially in orthotopic xenograft nude mice compared to non-stem cells [25]. To confirm the efficiency of regorafenib on the tumourigenic potential of GSC, we analysed the development of tumours in orthotopic xenograft nude mice with or without this molecule for 45 days. As shown in Figure 2A, regorafenib administered orally at daily doses of 30 mg/kg resulted in the inhibition of tumour growth. The size of the tumour was analysed using nestin immunostaining and was significantly lower in xenograft mice treated with regorafenib compared to untreated xenograft mice (4.37 ± 1.16 vs. 12.33 ± 3.02 mm^2^, *p* < 0.05). 

As regorafenib exhibits anti-angiogenic activity [15,16,18], we then assessed tumour vascularisation by performing CD31 staining. As expected, we noted a significant decrease in CD31+ vessels in tumours developed in xenograft mice treated with regorafenib compared to the untreated xenograft mice (101.4 ± 4.55 vs. 141.3 ± 14 CD31+ vessels/mm^2^, *p* < 0.05) (Figure 2B and Supplementary Figure S1). 

### 3.2. Regorafenib Inhibits Transdifferentiation of GSC In Vitro

**Figure 3:**
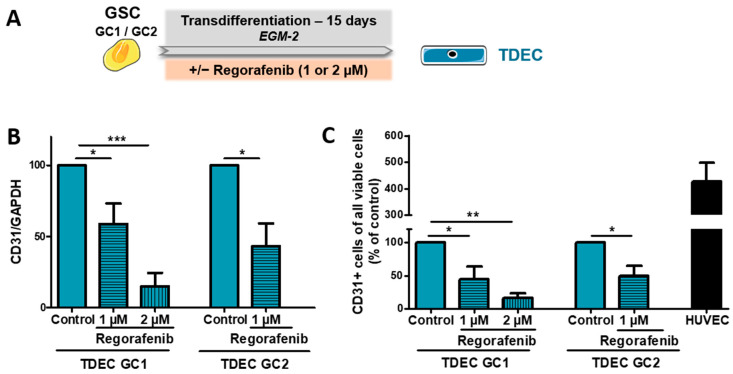

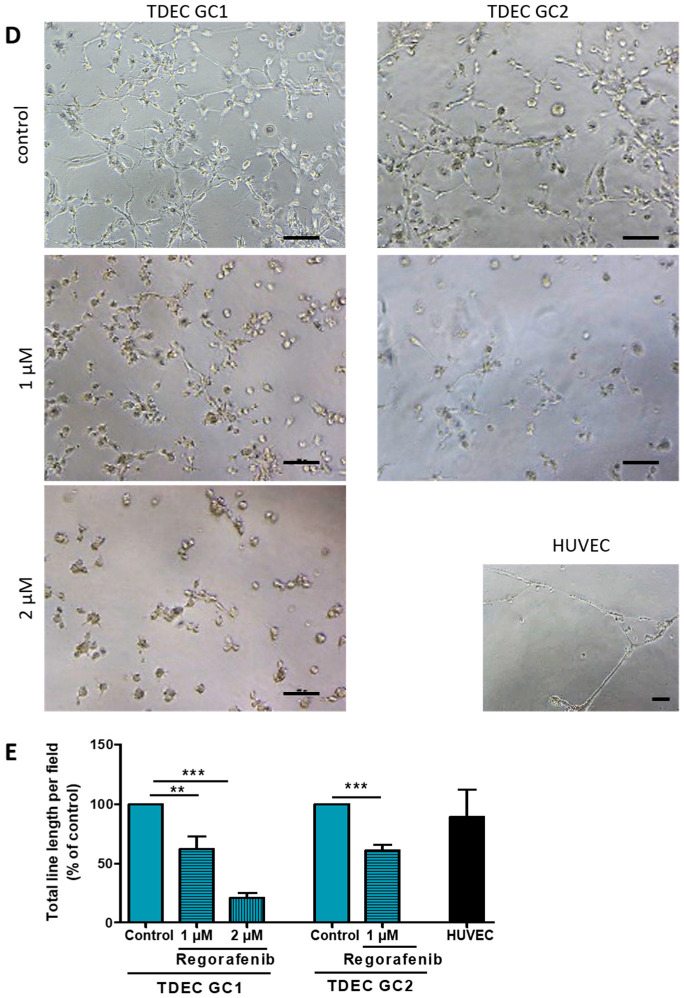
Regorafenib inhibits GSC transdifferentiation into TDEC in vitro. (**A**) GSC isolated from 2 patients (GC1 and GC2) were cultured in EGM-2 for 15 days in order to obtain TDEC (TDEC GC1 and TDEC GC2). (**B**) Immunoblot of CD31 in TDEC GC1 and TDEC GC2 with or without 1 or 2 µM of regorafenib. The graph shows the protein expression ratio normalised to TDEC obtained from each GSC and not treated with regorafenib (control). (**C**) Flow cytometric analysis of CD31 expression in TDEC obtained from GC1 and GC2 with or without regorafenib. The level of CD31 positive cells is expressed as the mean ± SEM normalised to TDEC obtained from each GSC and not treated with regorafenib (control). HUVEC is shown here as a positive control of CD31 expression. (**D**,**E**) Pseudotube formation assay. (**D**) Representative photographs of pseudotubes formed by TDEC GC1or TDEC GC2 treated or not with regorafenib. HUVEC is shown here as a positive control of pseudotube formation. Scale bars, 100 µm. (**E**) The graph shows the mean ± SEM of the total line length per field determined by quantification of at least 3 fields per well, normalised to TDEC obtained from each GSC and not treated with regorafenib (control). HUVEC is shown here as a positive control of pseudotube formation; * *p* < 0.05; ** *p* < 0.01; *** *p* < 0.001.

GBM are thus characterised by significant vascularisation potentially triggered by various mechanisms [9]. One of these mechanisms is the formation of vessels by tumour-derived endothelial cells (TDEC) following the transdifferentiation of GSC. As regorafenib clearly decreases GSC-mediated tumour formation in xenograft mice with a decrease in tumour vascularisation, we assumed that it could affect the transdifferentiation of GSC into TDEC. We therefore cultured our GSC (GC1 and GC2) in EGM-2 for 15 days with and without regorafenib before analysing the endothelial features of the TDEC obtained (Figure 3A). As 3 µM and 5 µM of regorafenib appear toxic to GSC after 7 days (Figure 1), we decided to test doses of 1 µM and 2 µM of regorafenib on transdifferentiation. We initially checked the effect of regorafenib on TDEC proliferation and thus viability after 6 days of culture. Regorafenib at 1 or 2 µM did not significantly influence the number of TDEC GC1 (Supplementary Figure S2). In TDEC GC2, 1 µM of regorafenib did not have a significant effect on cell number, whereas 2 µM significantly decreased the number of cells (50% decrease compared to the control). After 15 days of transdifferentiation, the number of TDEC GC2 cells treated with 2 µM of regorafenib was insufficient to perform the different experiments and to obtain a sufficient number of replicates. We therefore tested only the dose of 1 µM of regorafenib on TDEC GC2. 

We looked at AKT phosphorylation in order to verify the efficacy of 1 µM doses of regorafenib on TDEC GC1 and GC2 and 2 µM on TDEC GC1 (Supplementary Figures S3, S8 and S9 for original data). As regorafenib is a multi-kinase inhibitor, we decided to look at its impact on a signalling pathway protein that is common to the different tyrosine kinases targeted by this molecule. Doses of 1 and 2 µM of regorafenib both triggered a significant decrease in AKT phosphorylation in TDEC GC1 and TDEC GC2, which enabled us to conclude that the chosen doses were efficient. 

We therefore assessed the effect of regorafenib on the angiogenic features of TDEC GC1 and GC2. As shown in Figure 3B, 1 µM of regorafenib significantly decreased CD31 protein expression in TDEC GC1 and TDEC GC2 (see also Supplementary Figure S4 for a representative Western blot image and Figures S10 and S11 for original data). Moreover, 2 µM of regorafenib reduced CD31 protein expression in TDEC GC1 to an even greater extent compared to 1 µM (inhibition of 40% with 1 µM vs. 85% with 2 µM of CD31 protein expression in TDEC GC1, *p* < 0.05). Regorafenib also triggered a significant decrease in the number of CD31+ TDEC obtained from GC1 and GC2, as observed by FACS analysis (Figure 3C). Similarly, in TDEC GC1, we observed a dose-dependent effect with decreases of 56% and 85% in CD31+ cells with 1 µM and 2 µM of regorafenib, respectively. We then looked at the impact of regorafenib on the specific endothelial abilities of TDEC such as pseudotube formation in Matrigel^TM^. We also noted a significant decrease in pseudotube formation of both TDEC in the presence of regorafenib. The result was dose-dependent in TDEC GC1 (Figure 3D,E).

### 3.3. Regorafenib Inhibits Irradiation (IR)-Induced Transdifferentiation of GSC In Vitro

**Figure 4:**
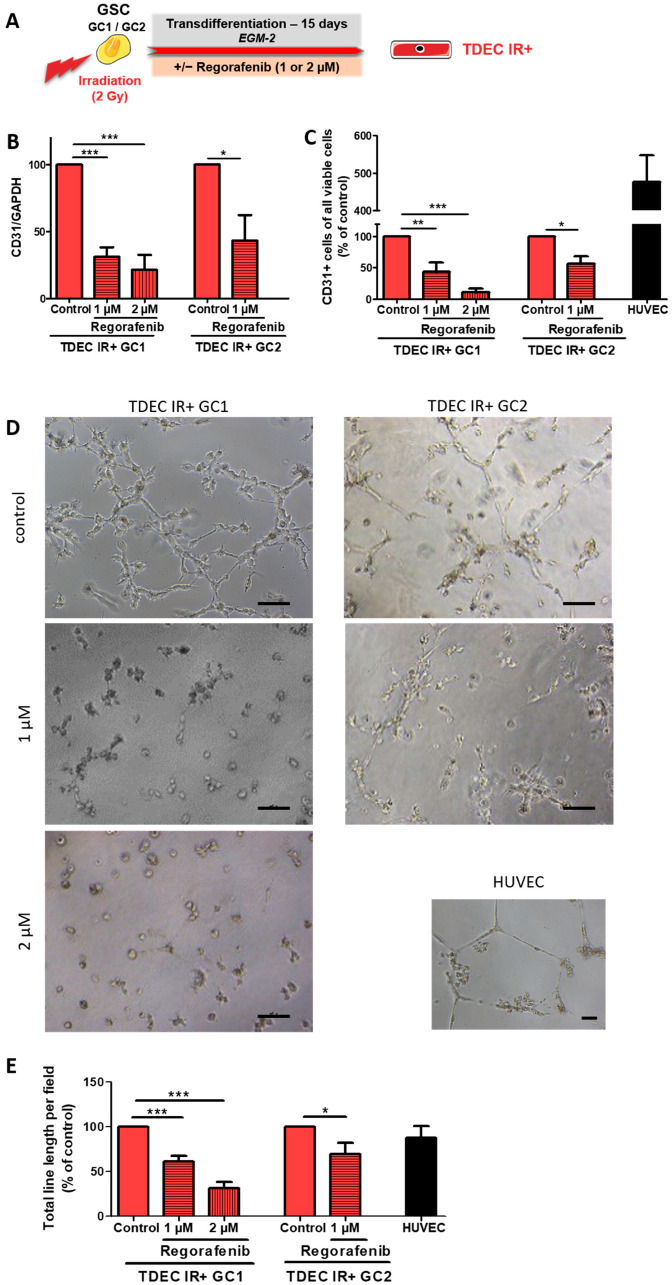
Regorafenib inhibits irradiation-induced transdifferentiation in vitro. (**A**) GSC isolated from 2 patients (GC1 and GC2) were irradiated (2Gy) and then cultured in EGM-2 for 15 days in order to obtain TDEC IR+ (TDEC IR+ GC1 and TDEC IR+ GC2). (**B**) Immunoblot of CD31 in TDEC IR+ GC1 and TDEC IR+ GC2 with or without 1 or 2 µM of regorafenib. The graph shows the protein expression ratio normalised to TDEC IR+ obtained from each GSC without regorafenib (control). (**C**) Flow cytometric analysis of CD31 expression in TDEC IR+ obtained from GC1 and GC2, with or without regorafenib. The level of CD31 positive cells is expressed as the mean ± SEM normalised to TDEC IR+ obtained from each GSC without regorafenib (control). HUVEC is shown here as a positive control of CD31 expression. (**D**,**E**) Pseudotube formation assay. (**D**) Representative photographs of pseudotubes formed by TDEC IR+ GC1or TDEC IR+ GC2 treated or not with regorafenib. HUVEC is shown here as a positive control of pseudotube formation. Scale bars, 100 µm. (**E**) The graph shows the mean ± SEM of the total line length per field determined by quantification of at least 3 fields per well normalised to TDEC IR+ obtained from each GSC and not treated with regorafenib (control). HUVEC is shown here as a positive control of pseudotube formation; * *p* < 0.05; ** *p* < 0.01; *** *p* < 0.001.

We and others previously showed that IR potentiates transdifferentiation of GSC both in vitro and in vivo [13,14]. Notably, we showed that the Tie2 signalling pathway was involved in IR-induced transdifferentiation of GSC [14]. As regorafenib also inhibits Tie2 signalling, we decided to assess its impact on IR-induced transdifferentiation. The two different GSC were subjected to clinical 2-Gy IR and were placed in EGM2 with or without regorafenib for 15 days (Figure 4A). As previously, we looked at the impact of regorafenib on TDEC proliferation 6 days after the onset of transdifferentiation and observed that TDEC IR+ were sensitive to regorafenib (Supplementary Figure S5). In fact, the number of TDEC IR+ GC1 treated with 1 or 2 µM of regorafenib was significantly lower than the number of TDEC IR+ GC1 controls with a dose-dependent effect. However, the decrease in live cells was less than 50% with the two different doses tested. Thus, we decided to assess the impact on the TDEC IR+ GC1 endothelial features of both doses. Regarding the TDEC IR+ GC2, the number of cells treated with 1 µM of regorafenib did not differ significantly from that of untreated TDEC IR+ GC2, whereas the 2 µM dose triggered a significant 65% decrease in the number of live cells. As previously, after 15 days of transdifferentiation, the number of TDEC IR+ GC2 cells treated with 2 µM of regorafenib was insufficient to perform the different experiments. Therefore, we were only able to assess the dose of 1 µM of regorafenib on TDEC IR+ GC2. After checking that the doses of 1 µM and 2 µM of regorafenib were actually effective in TDEC IR+ (Supplementary Figures S6, S8 and S9 for original data), we tested their impact on TDEC IR+ endothelial characteristics. In terms of CD31 protein expression in TDEC IR+ GC1, 1 µM and 2 µM of regorafenib both triggered a significant decrease in its expression (Figure 4B; see also Supplementary Figure S4 for a representative Western blot image and Figures S10 and S11 for original data). Similarly, in TDEC IR+ GC2, CD31 protein expression was significantly reduced after treating with 1 µM of regorafenib for 15 days. 

Since regorafenib was seen to significantly inhibit TDEC IR+ proliferation, we then looked at the CD31+ live cells using flow cytometry. As shown in Figure 4C, the number of TDEC IR+ GC1 CD31+ live cells was clearly reduced in a dose-dependent manner in the presence of regorafenib (43.66 ± 14.22% and 10.54 ± 5.57% of TDEC IR+ expressed CD31 after treatment with 1 or 2 µM of regorafenib, respectively, compared to the TDEC IR+ control) (Figure 4C). One micromolar of regorafenib also significantly reduced the number of TDEC IR+ GC2 CD31+ (inhibition of 44%). Regarding pseudotube formation by TDEC IR+ GC1, both doses of regorafenib triggered a significant decrease (Figure 4D,E). This inhibition was similar to that observed in TDEC GC1 without irradiation (Figure 3E). Regorafenib therefore appears to effectively impact TDEC GC1 pseudotube formation as in the case of TDEC IR+ GC1. One micromolar of regorafenib triggered a significant decrease in pseudotube formation in TDEC IR+ GC2. However, although this decrease seems slightly lower than that observed in TDEC GC2 without irradiation (Figure 3E), it is not significantly different (31% of inhibition in TDEC IR+ GC2 vs. 40% inhibition in TDEC GC2, *p* = 0.51).

### 3.4. High-Dose Regorafenib Inhibits the Tie2 Signalling Pathway in TDEC IR+ In Vitro

**Figure 5:**
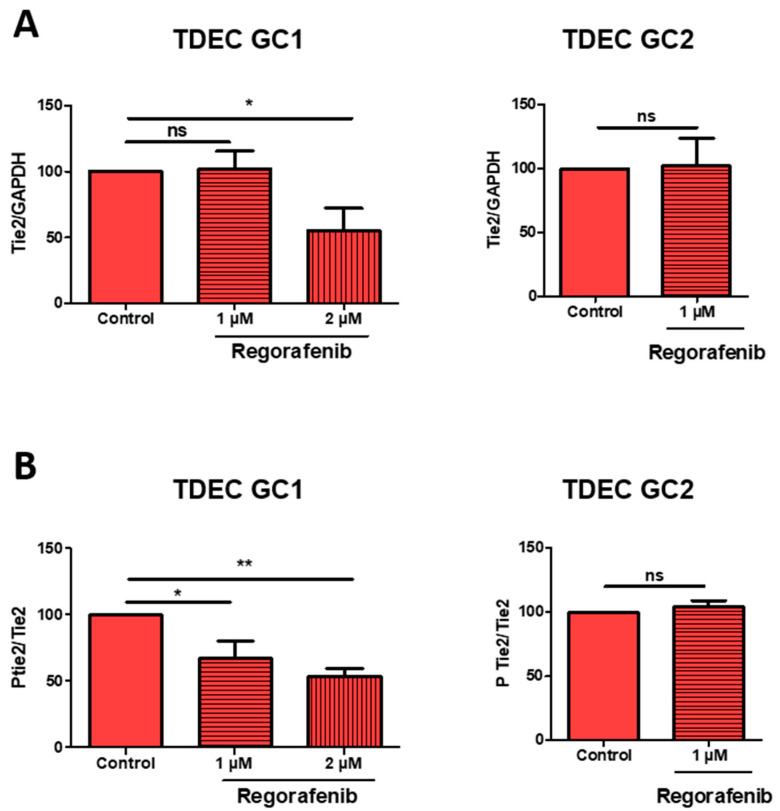
High-dose regorafenib decreases Tie expression and phosphorylation in TDEC IR+. (**A**) Immunoblot of Tie2 in TDEC IR+ GC1 and TDEC IR+ GC2 with or without 1 or 2 µM of regorafenib. Blots were quantified and the graphs show the protein expression ratio normalised to TDEC IR+ obtained from each GSC without regorafenib (control). (**B**) Ptie2/Tie expression ratio evaluated by immunoblot. The graphs show the protein expression ratio normalised to TDEC IR+ obtained from each GSC without regorafenib (control); * *p* < 0.05; ** *p* < 0.01.

As we previously showed that IR-induced transdifferentiation is partly driven by activation of the Tie2 signalling pathway, we examined the impact of regorafenib on this pathway in TDEC IR+ GC1 and GC2 [14]. As shown in Figure 5A,B, 1 µM of regorafenib did not trigger a significant decrease in Tie2 expression in TDEC IR+ GC1 and GC2, whereas the dose of 2 µM in TDEC IR+ GC1 significantly reduced the expression of Tie2 as well as its phosphorylation (see also Supplementary Figures S12 and S13 for original data). One micromolar of regorafenib also significantly inhibited Tie2 phosphorylation in GC1 but not in GC2. We showed previously that Tie2 is not the main driver in transdifferentiation of non-irradiated GSC [14]. We highlighted here that regorafenib does not reduce Tie2 activation in TDEC without irradiation and that only 2 µM of regorafenib decreases Tie2 expression in GC1 (Supplementary Figures S7, S12 and S13). We can thus conclude that, provided it is not overly toxic on cell proliferation, high-dose regorafenib inhibits the IR-induced transdifferentiation of GSC by inhibiting the Tie2 signalling pathway.

### 3.5. Regorafenib Inhibits Classical and IR-Induced Transdifferentiation In Vivo

**Figure 6:**
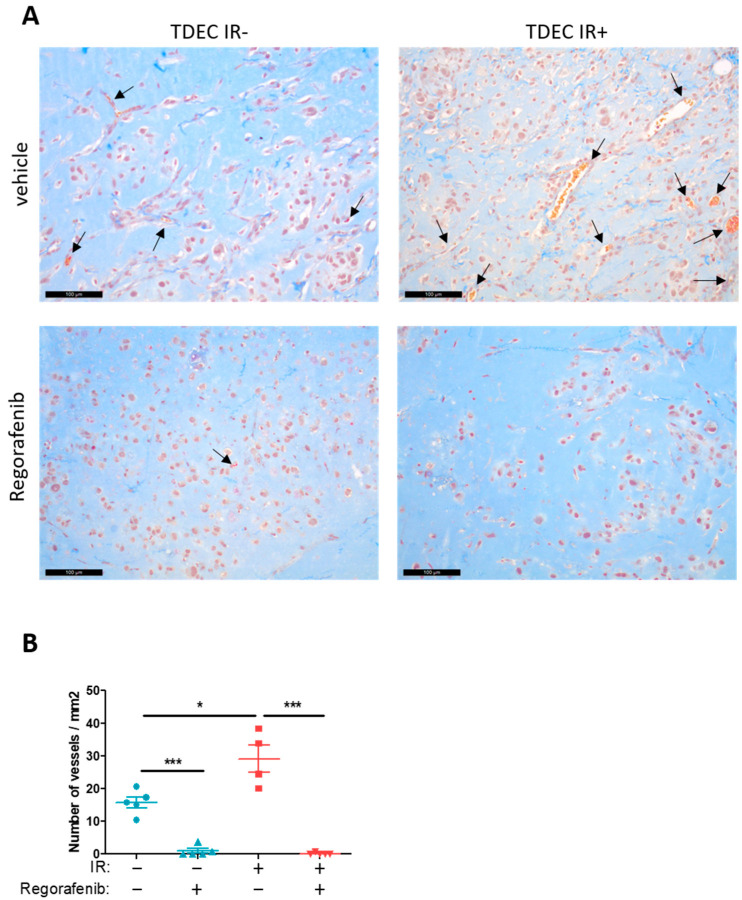
Regorafenib inhibits GSC-mediated conventional and irradiation-induced transdifferentiation in vivo. Matrigel^TM^ plug assay. (**A**) Representative trichrome Masson sections of Matrigel plugs with TDEC obtained from non-irradiated GC1 (left photos) or irradiated GC1 (right photos) with (lower panel) or without (upper panel) regorafenib. The black arrows indicate functional blood vessels. Scale bars, 100 µm. (**B**) Quantification of functional blood vessels in Matrigel TM plugs/mm^2^. The number of vessels/mm^2^ was expressed as the mean ± SEM of 5 mice for untreated TDEC IR-, TDEC IR- treated with regorafenib and TDEC IR+ treated with regorafenib and of 4 untreated TDEC IR+ mice; * *p* < 0.05; *** *p* < 0.001.

To confirm the impact of regorafenib on transdifferentiation, we performed the Matrigel plug assay using TDEC GC1 obtained from non-irradiated and irradiated GC1. Following subcutaneous implantation of the plugs containing the cells, regorafenib or the vehicle was administered orally by gavage for 14 days. Interestingly, plugs with TDEC IR- of mice treated with regorafenib had significantly fewer functional blood vessels than plugs with the TDEC IR- control (mean: 0.9 ± 0.73 vs. 15.83 ± 1.68 vessels/mm^2^, *p* < 0.001) (Figure 6). As previously shown [14], we found more functional blood vessels in plugs with the TDEC IR+ control compared to plugs with the TDEC IR- control (mean: 29.17 ± 4.2 vs. 15.83 ± 1.68 vessels/mm^2^, *p* < 0.05). Remarkably, as seen earlier, plugs with TDEC IR+ treated with regorafenib had significantly fewer functional blood vessels than plugs with the TDEC IR+ control (mean: 0.15 ± 0.15 vs. 29.17 ± 4.2 vessels/mm^2^, *p* < 0.001). 

All of these results suggest that regorafenib is an important inhibitor of classical and IR-induced transdifferentiation both in vitro and in vivo.

## 4. Discussion

Despite the fact that GBM are the most common and lethal primary brain tumours in adults, the standard of care for patients has been the same for more than a decade. Indeed, the combination of chemotherapy with temozolomide and radiotherapy (30 daily fractions of 2 Gy) resulted in an increase in median survival from 12.1 to 14.6 months [2,3]. However, relapse is mostly inevitable. Various new molecular combinations have been tested in numerous clinical trials in an attempt to improve GBM outcome. In the randomised, open-label, phase II REGOMA trial, Lombardi et al. recently showed that regorafenib, a multiple kinase inhibitor, significantly improved overall survival (median OS: 7.4 vs. 5.6 months; *p* = 0.0009) and 6-month progression free survival (PFS): 16.9% vs. 8.3%; *p* = 0.022) compared to conventional lomustine treatment in patients with relapsed glioblastoma [19]. Prior to this clinical trial, preclinical studies assessing the effect of regorafenib on solid malignancies, and notably brain tumours, had already been performed [16,18] but never on glioblastoma stem cells (GSC). In fact, GSC are both radio-resistant and chemoresistant, which may account for the therapeutic resistance of these aggressive tumours [26]. Finding molecules to target and kill these resistant cells should improve progression-free survival and thus overall survival. Our work shows for the first time that regorafenib significantly inhibits GSC neurosphere formation in a dose-dependent manner. This observation could be due to the blockage by regorafenib of tyrosine kinase involved in GSC proliferation. In fact, GSC neurosphere formation requires two growth factors, namely EGF and FGF, both of which activate tyrosine kinase receptors, EGFR and FGFR, respectively [27]. The inhibition of FGFR1 and FGFR2 by regorafenib has already been described, but there has been no mention of EGFR inhibition [15,16]. Nevertheless, regorafenib also targets protein kinases such as RAF-1 and BRAF, both of which are involved in downstream EGFR signalling [28]. Further experiments are required to confirm the way in which regorafenib reduces GSC neurosphere formation. Interestingly, Gouaze-Andersson et al. showed that FGFR1 is a key regulator in GSC radioresistance [23]. As regorafenib targets FGFR1, it would be interesting to explore the impact of combined regorafenib and irradiation therapy on GSC radioresistance in vitro and in vivo. We confirmed the effect of regorafenib on GSC using the orthotopic xenograft model. Regorafenib significantly inhibits GSC tumour formation in nude mice. The effect observed could be due to the direct impact on GSC and/or the inhibition of blood vessel formation. Indeed, regorafenib inhibits various tyrosine kinase receptors involved in vascularisation such as VEGFR, Tie2 and PDGFR [15]. Moreover, Daudigeos-Dubus et al. showed that the in vivo antitumour activity of regorafenib was mediated in part by anti-angiogenic effects [18]. In keeping with this report, we show that regorafenib-treated xenografts reduced the number of blood vessels compared to control tumours, as evidenced by CD31 staining. 

Different mechanisms of glioma-associated vascularisation have been described in GBM such as conventional mechanisms of angiogenesis, vasculogenesis and vascular mimicry [9]. The VEGF/VEGFR signalling pathway plays a prominent role in all three mechanisms [29,30,31,32]. It is not surprising then to witness inhibition of these mechanisms by regorafenib, since it inhibits VEGFR kinase activity with a low IC50 (mean ± SD: 3 nM ± 2) [15,16]. GSC transdifferentiation into endothelial cells is another mechanism for the formation of new blood vessels in GBM [10,11,12,33]. The signalling pathways involved in this mechanism are not clearly defined. In fact, some studies show that the VEGF signalling pathway was at least partly implicated, whereas others show that transdifferentiation was VEGF-independent, which could explain GBM resistance to anti-angiogenic therapies [11,12,34,35]. As regorafenib also inhibits many other tyrosine kinase receptors, we investigated its effect on GSC transdifferentiation. Regorafenib clearly reduced the TDEC count as well as their pro-angiogenic abilities both in vitro and in vivo. As we and others have previously shown that irradiation increases the number of TDEC and potentiates the pro-angiogenic features of TDEC, we also looked at the effect of regorafenib on TDEC IR+ and observed the same results: a decrease in CD31 expression and inhibition of pro-angiogenic abilities of TDEC IR+ in vitro and in vivo [13,14]. Although the Tie2 signalling pathway is a key player in IR-induced potentiation of the pro-angiogenic features of TDEC IR+, only a high dose of regorafenib, i.e., 2 µM, decreased Tie2 expression and phosphorylation, whereas 1 and 2 µM decreased TDEC pro-angiogenic features. Other signalling pathways may therefore be involved in IR-induced transdifferentiation, and further experiments are required to unveil the inner workings of this mechanism. 

## 5. Conclusions

Overall, regorafenib appears to inhibit tumour formation as well as the formation of new blood vessels via GSC transdifferentiation into TDEC. This finding is of major importance, since one study using a three-dimensional mathematical model reported that combination therapy comprising current standard treatments such as radiotherapy, temozolomide and bevacizumab with treatment to target GSC transdifferentiation could reduce tumour invasiveness and size and ultimately lead to tumour suppression [36]. These in silico data are finally consistent with the results of the phase II clinical trial, REGOMA, that highlighted the superiority of regorafenib versus lomustine in patients with relapsed glioblastoma [19]. Moreover, considering the impact of regorafenib on transdifferentiation, as shown in this study, new therapeutic strategies in recently diagnosed GBM patients combining radiotherapy/temozolomide and regorafenib, which is already currently used in the treatment of metastatic colorectal cancer and gastrointestinal stromal tumours, could be of major interest. Indeed, an open-label, randomised, phase II/III multi-arm platform trial (GBM AGILE) assessing the efficiency of regorafenib in newly diagnosed and recurrent GBM is ongoing, and the results are long awaited [37].

## Data Availability

The data presented in this study are available on request from the corresponding author.

## Supplementary Materials

The following supporting information can be downloaded at: https://www.mdpi.com/article/10.3390/cancers14061551/s1, Figure S1: Representative scans of Nestin (upper panel) and CD31 staining (median panel) of mice #2 and #5; Figure S2: Regorafenib impact on TDEC proliferation; Figure S3: Efficiency of regorafenib on TDEC; Figure S4: CD31 protein levels measured by western-blot in TDEC obtained from GSC, with or without irradiation, and with or without regorafenib; Figure S5: Regorafenib impact on TDEC IR+ proliferation; Figure S6: Efficiency of regorafenib on TDEC IR+; Figure S7: Regorafenib impact on Tie2 expression and phosphorylation in TDEC; Figure S8: Raw data for AKT and PAKT western blots of GC1; Figure S9: Raw data for AKT and PAKT western blots of GC2; Figure S10: Raw data for CD31 western blots of GC1; Figure S11: Raw data for CD31 western blots of GC2; Figure S12: Raw data for Tie2 and Ptie2 western blots of GC1; Figure S13: Raw data for Tie2 and Ptie2 western blots of GC2.
