# Supplementary material for: Impact of Regorafenib on Endothelial Transdifferentiation of Glioblastoma Stem-like Cells
Source: Cancers (Basel). 2022 Mar 18;14(6):1551. doi: 10.3390/cancers14061551 (PMC8946617; doi:10.3390/cancers14061551)
Supplement: Supplementary file 1 [file cancers-14-01551-s001.zip › cancers-1574500-supplementary.pdf]

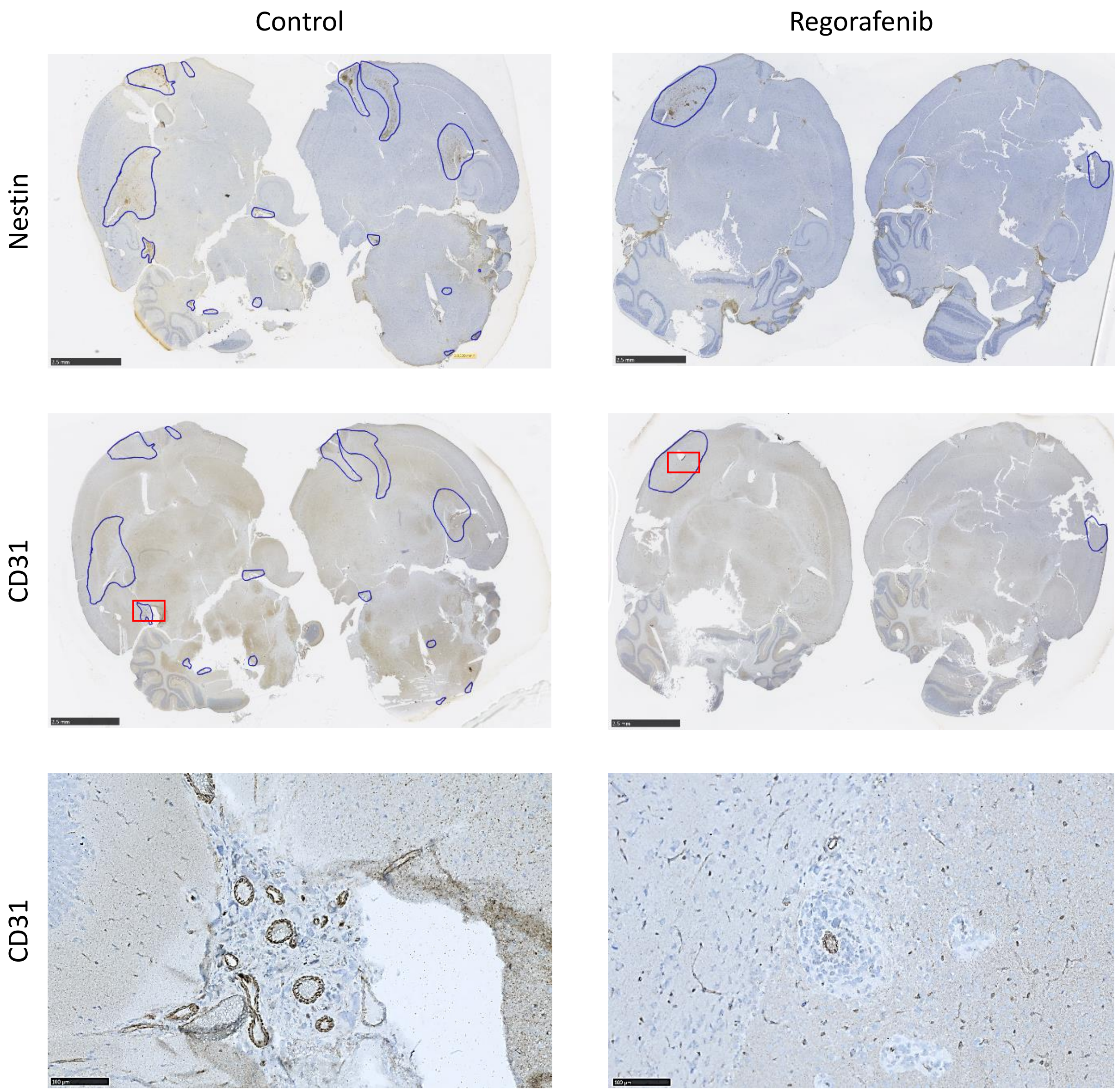

Supplementary Figure S1: Representative scans of Nestin (upper panel) and CD31 staining (median panel) of mice #2 and #5 whole brains. Tumour areas are circled in blue using nestin staining. These areas were reported on CD31 staining brains to determine the tumoral zone in which photographs of CD31+ blood vessels were taken. The red rectangles correspond to the area showed in the lower panel at higher magnification (lower panel) as well as in figure 2B (mice #2 and #5). Scale bars upper and median panel 2,5mm and lower panel 100µm.

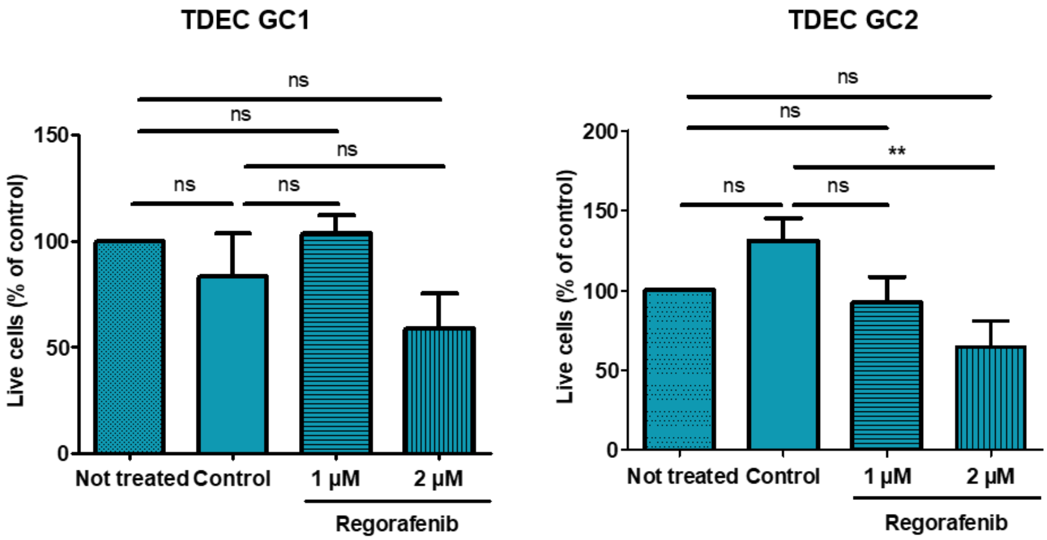

Supplementary Figure S2: Regorafenib impact on TDEC proliferation

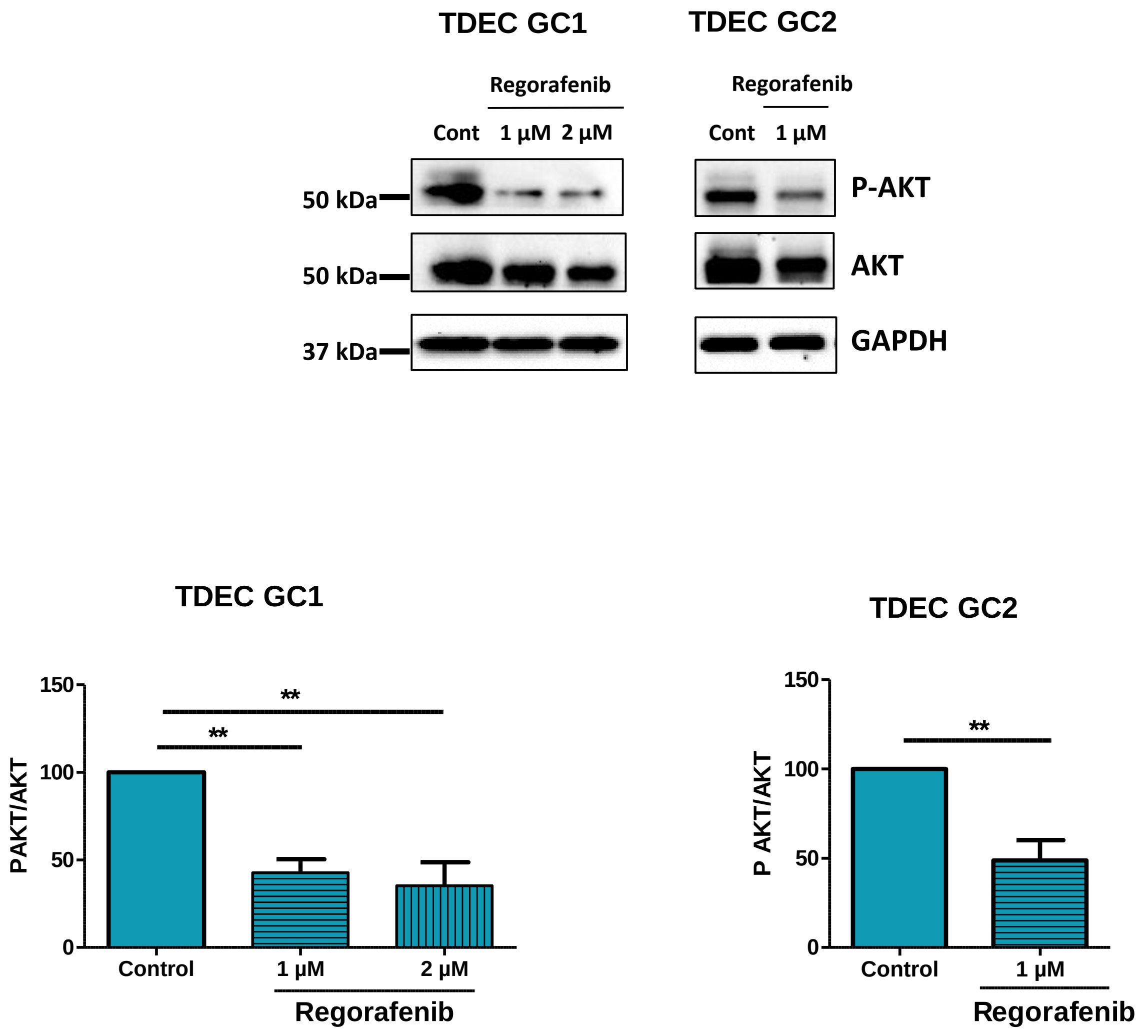

Supplementary Figure S3: Efficiency of regorafenib on TDEC

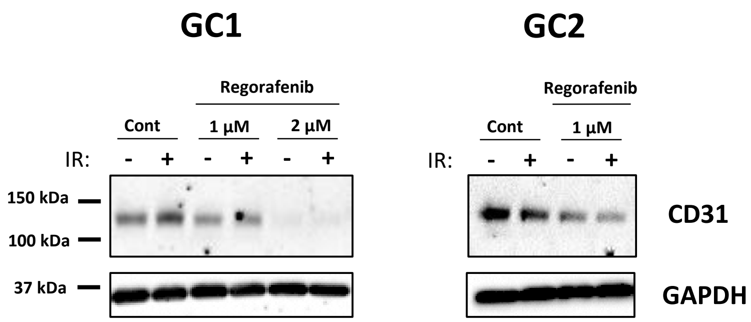

Supplementary Figure S4: CD31 protein levels measured by western-blot in TDEC obtained from GSC, with or without irradiation, and with or without regorafenib

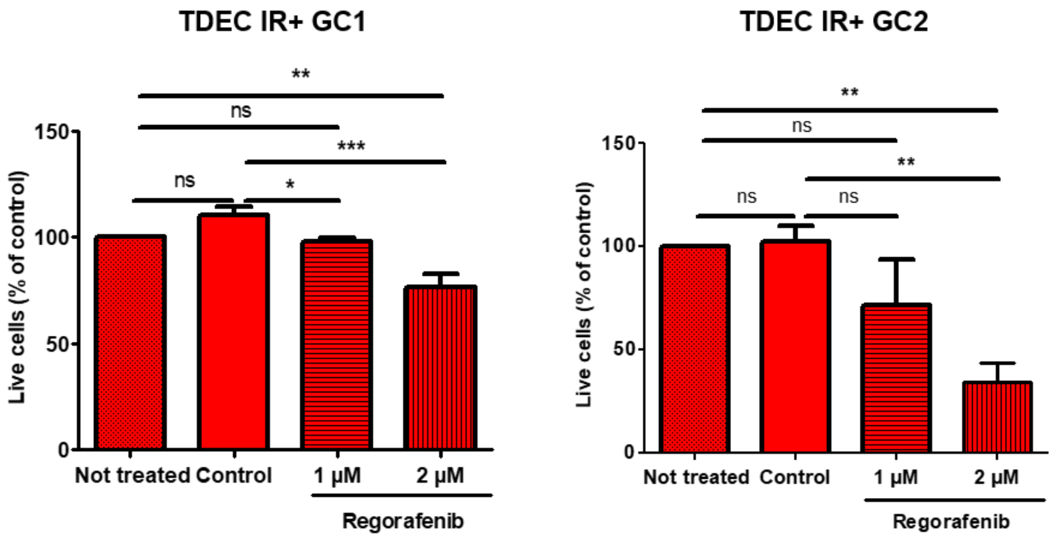

Supplementary Figure S5: Regorafenib impact on TDEC IR+ proliferation

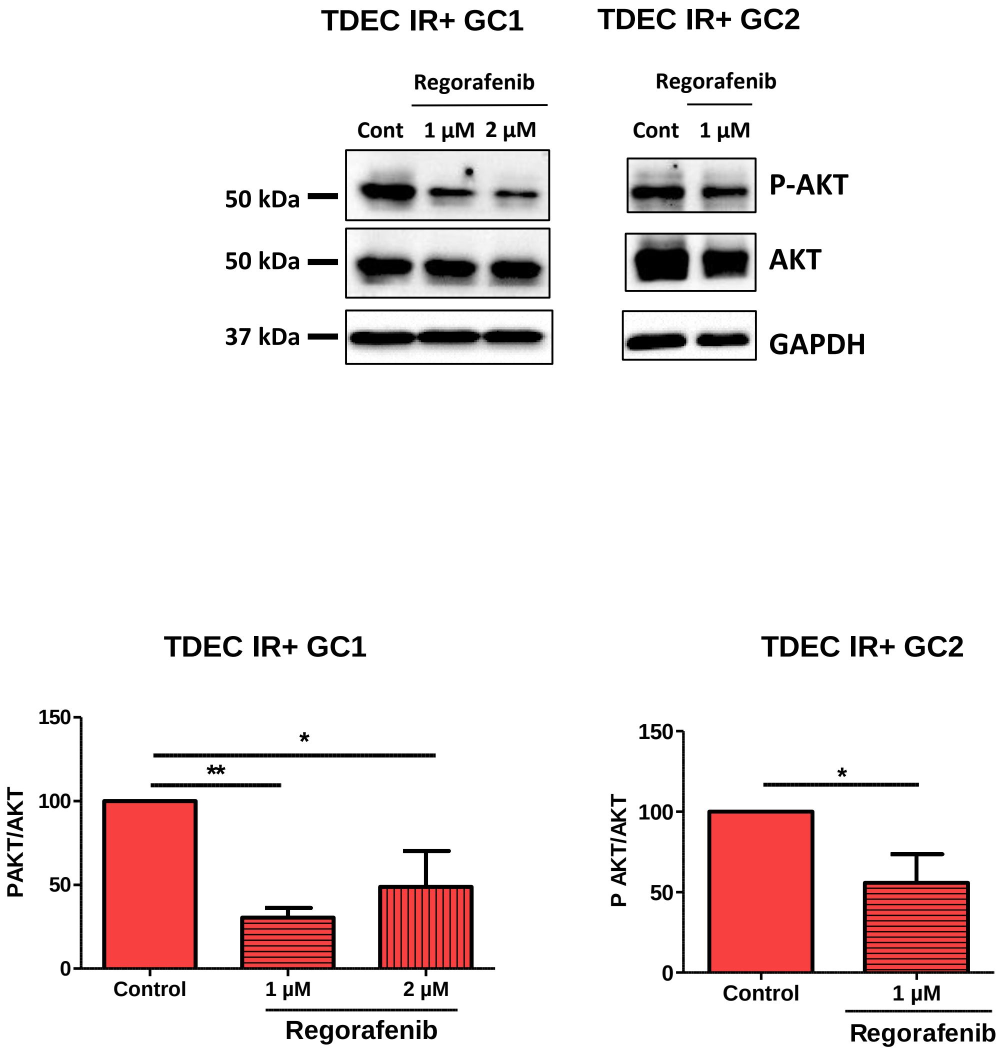

Supplementary Figure S6: Efficiency of regorafenib on TDEC IR+

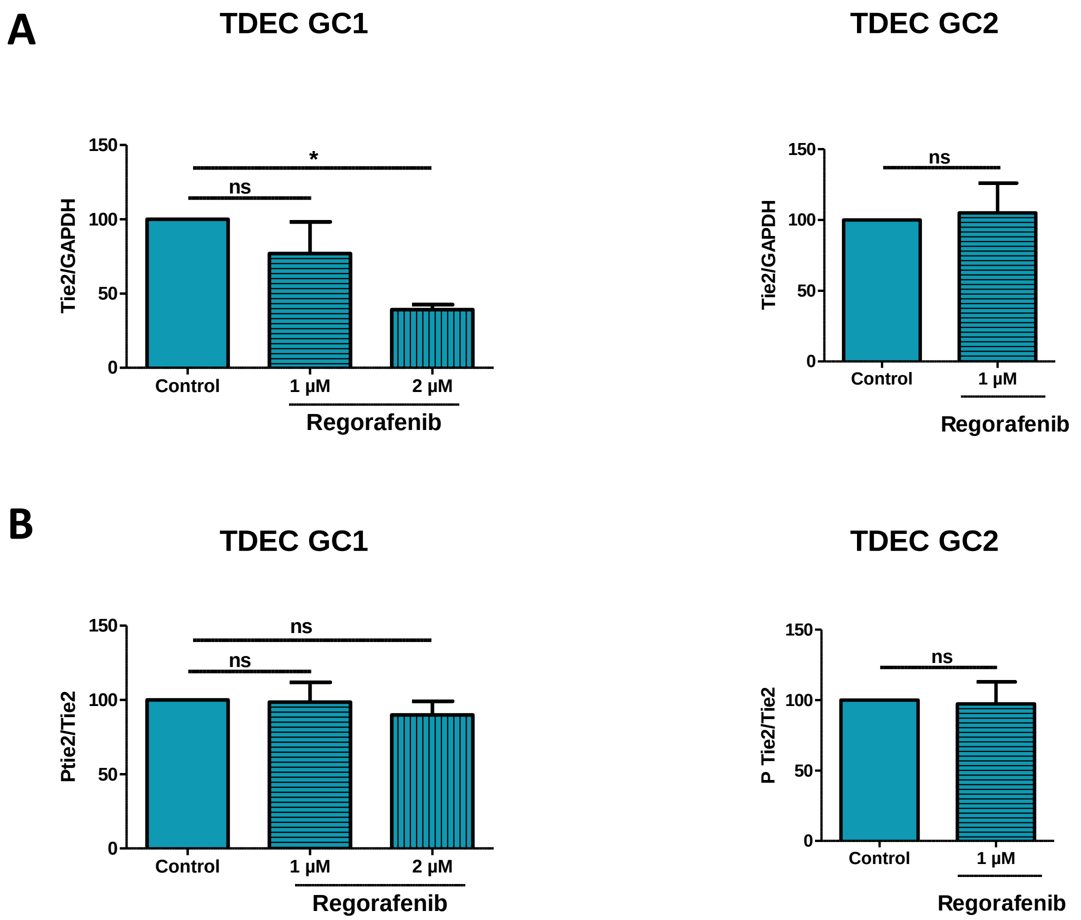

Supplementary Figure S7: Regorafenib impact on Tie2 expression and phosphorylation in TDEC

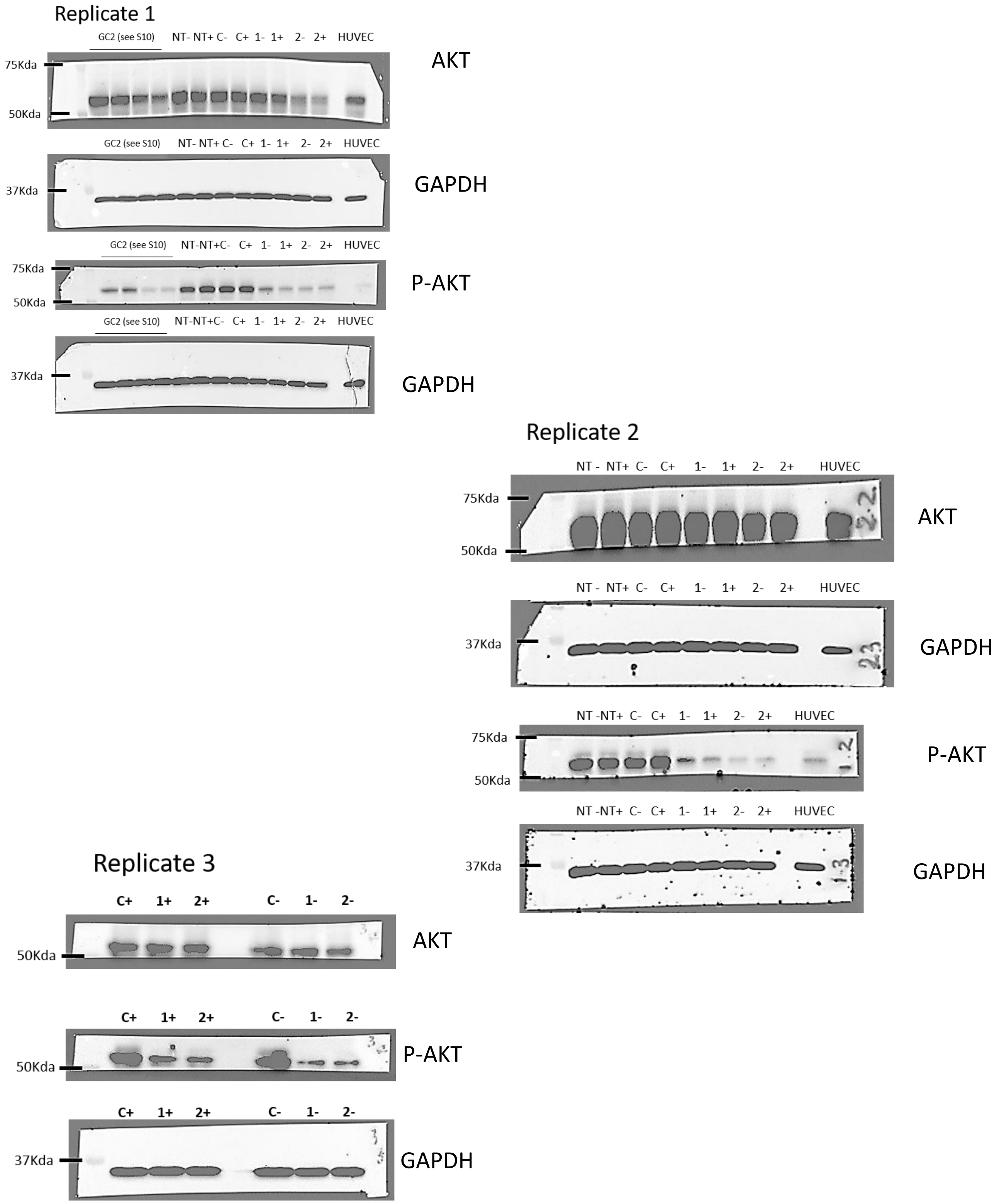

Supplementary Figure S8: Raw data for AKT and PAKT western blots of GC1. Figures S3 and S6 represent the quantification of 3 replicates shown here. Replicate 3 is raw data for figure presented in S3 and S6. Ladder markers sizes shown on left. AKT expected molecular weight/size is 60 kDa. P-AKT expected molecular weight/size is 60 kDa. GAPDH expected molecular weight/size is 36 kDa. *NT: not treated; C: control; 1: 1 µM of regorafenib; 2: 2 µM of regorafenib; -: not irradiated; +: irradiated.*

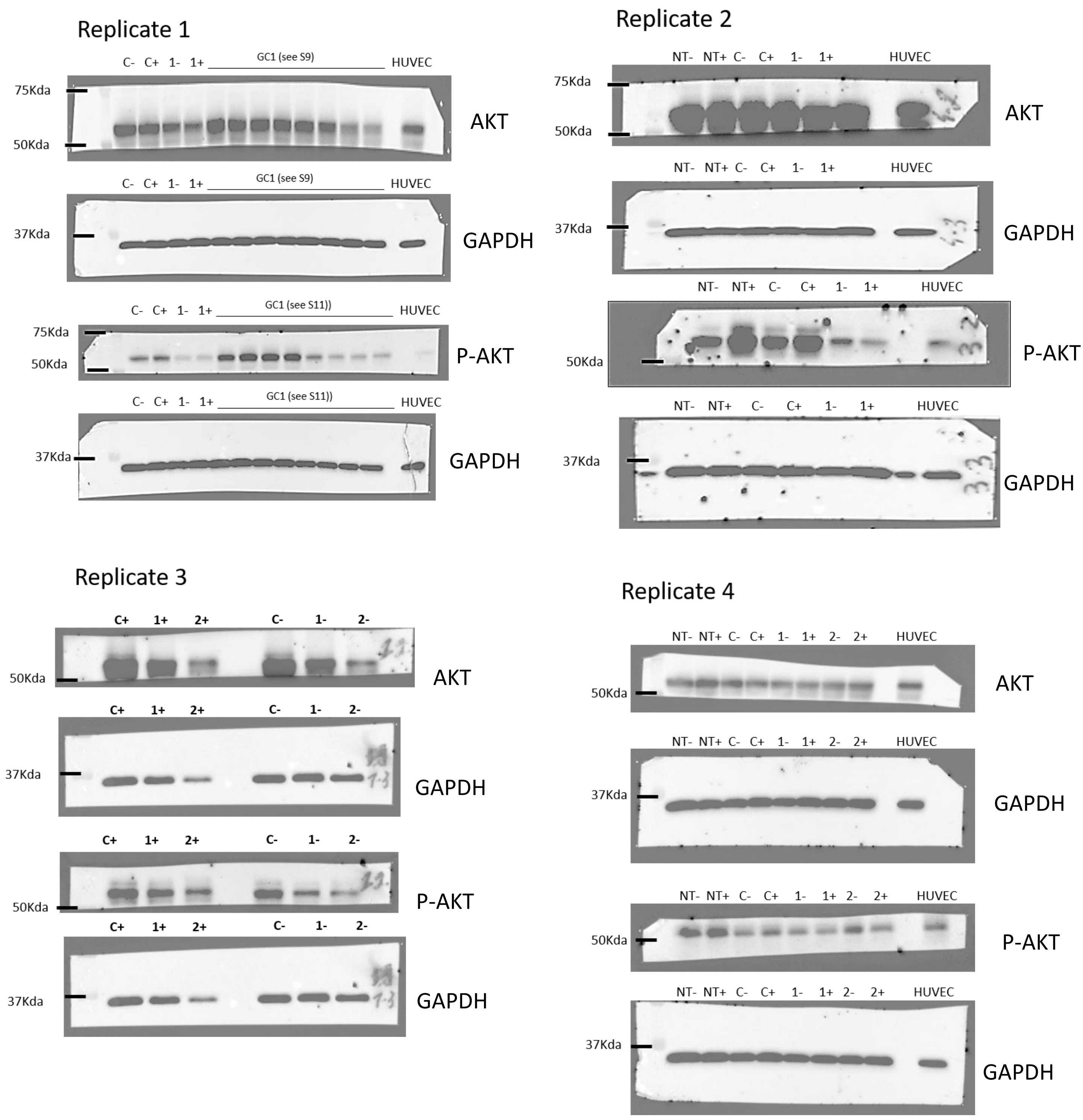

Supplementary Figure S9: Raw data for AKT and PAKT western blots of GC2. Figures S3 and S6 represent the quantification of 3 replicates shown here. Replicate 3 is raw data for figure presented in S3 and S6. Ladder markers sizes shown on left. AKT expected molecular weight/size is 60 kDa. P-AKT expected molecular weight/size is 60 kDa. GAPDH expected molecular weight/size is 36 kDa. *NT: not treated; C: control; 1: 1 µM of regorafenib; 2: 2 µM of regorafenib; -: not irradiated; +: irradiated.*

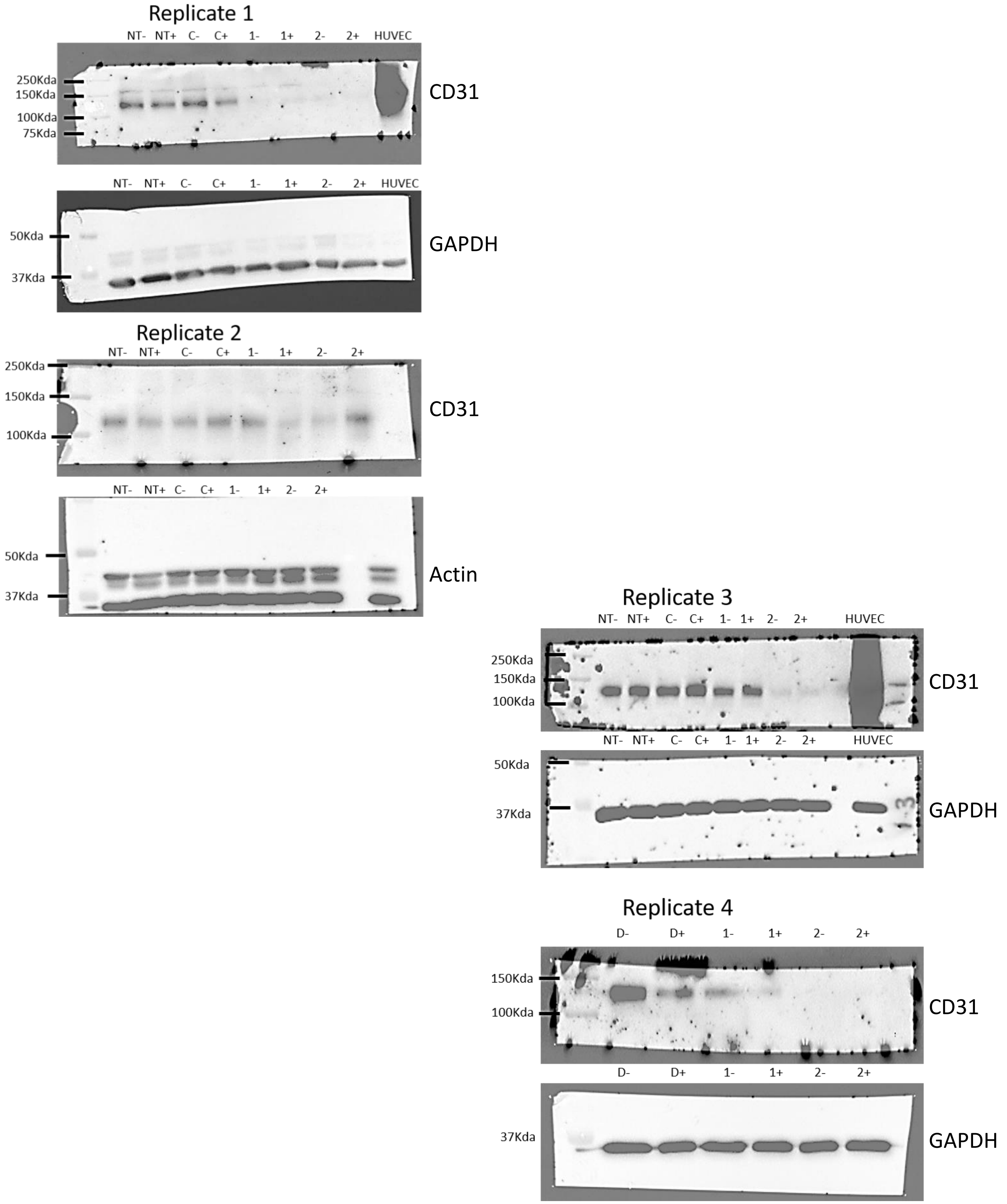

Supplementary Figure S10: Raw data for CD31 western blots of GC1. Figures 3B and 4B represent the quantification of 4 replicates shown here. Replicate 3 is raw data for figure presented in S4. Ladder markers sizes shown on left. CD31 expected molecular weight/size is 130 kDa. GAPDH expected molecular weight/size is 36 kDa. Actin expected molecular weight/size is 43 kDa.. *NT: not treated; C: control; 1: 1 µM of regorafenib; 2: 2 µM of regorafenib; -: not irradiated; +: irradiated.*

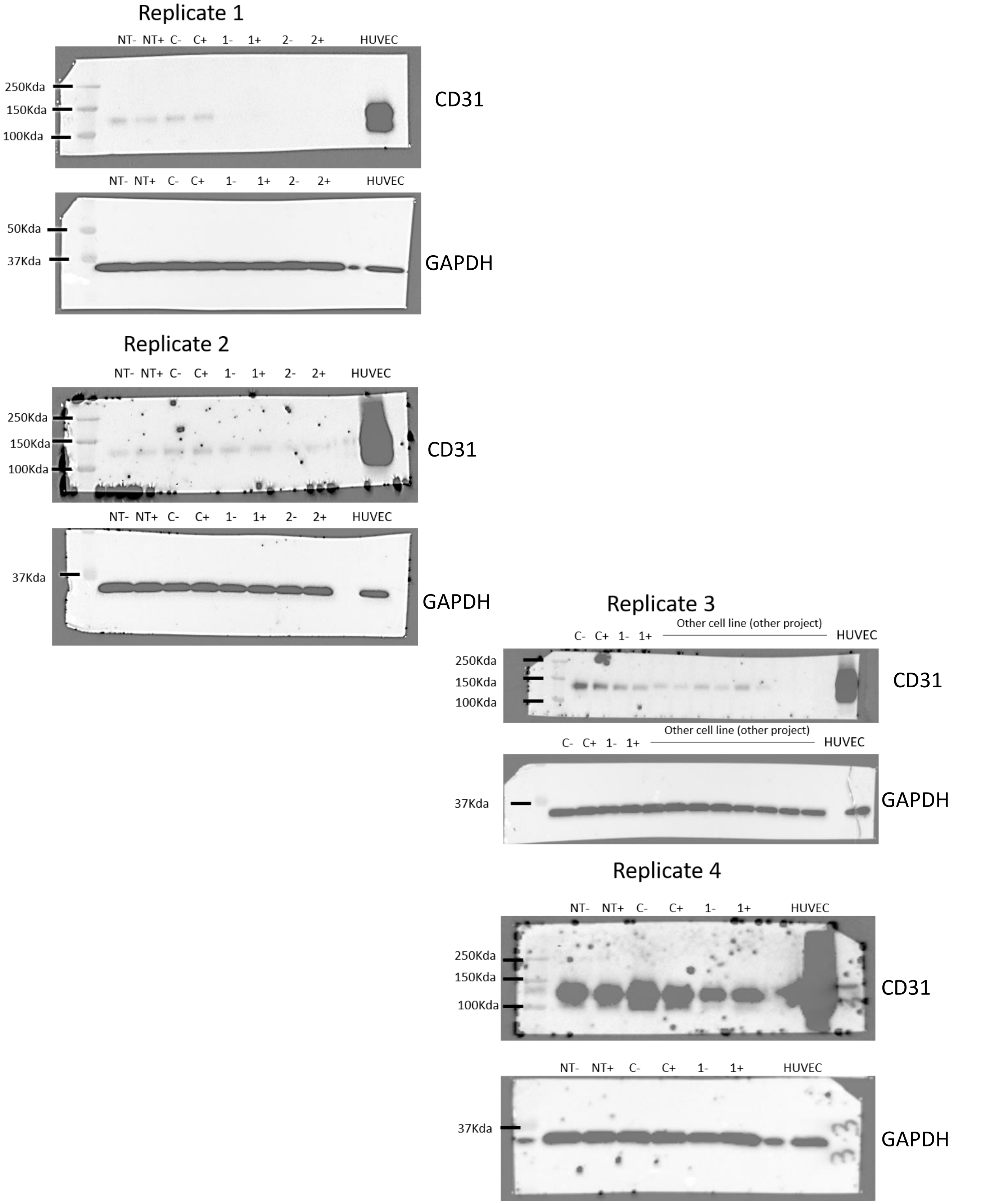

Supplementary Figure S11: Raw data for CD31 western blots of GC2. Figures 3B and 4B represent the quantification of 4 replicates shown here. Replicate 3 is raw data for figure presented in S4. Ladder markers sizes shown on left. CD31 expected molecular weight/size is 130 kDa. GAPDH expected molecular weight/size is 36 kDa. *NT: not treated; C: control; 1: 1 μM of regorafenib; 2: 2 μM of regorafenib; -: not irradiated; +: irradiated.*

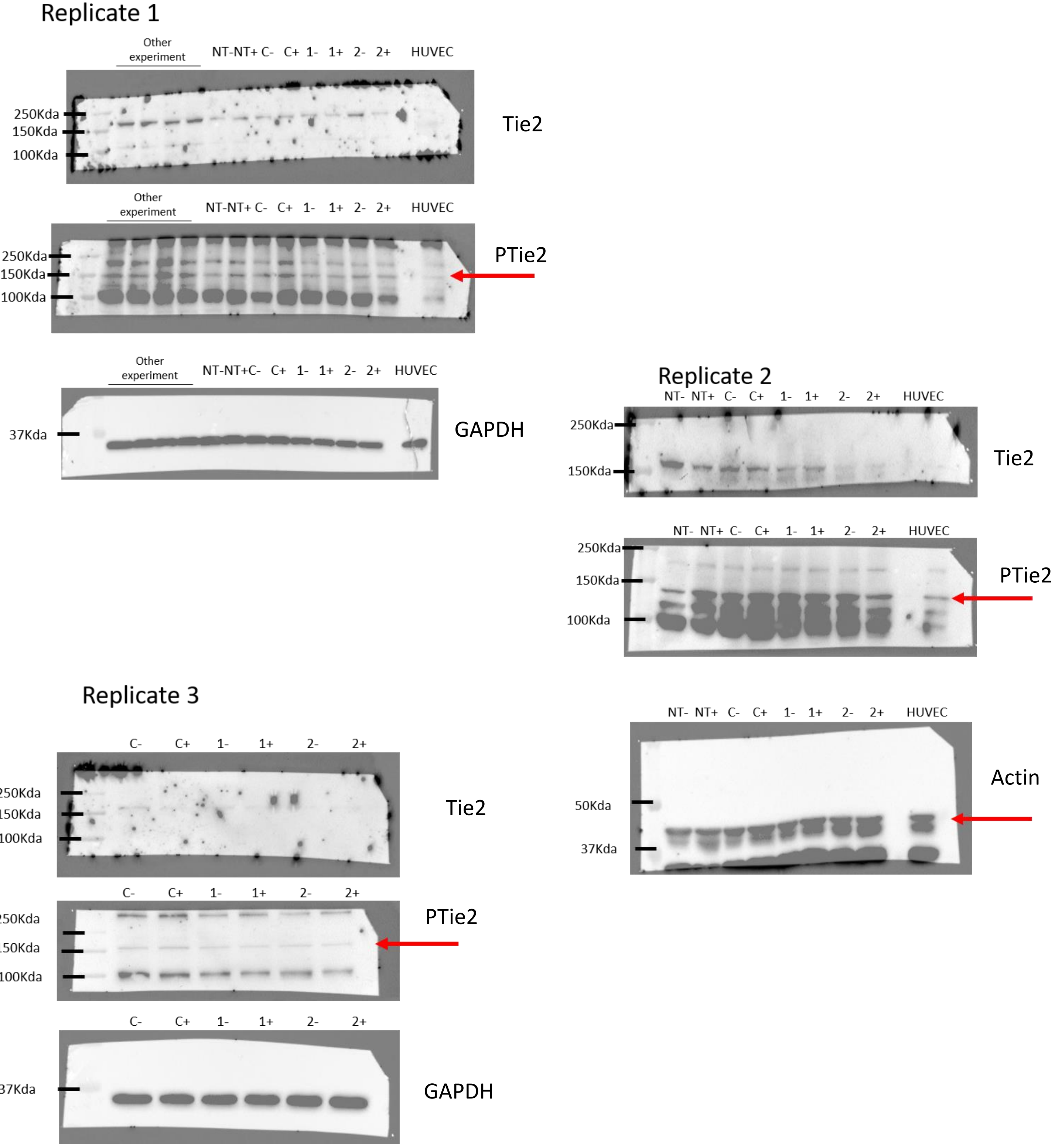

Supplementary Figure S12: Raw data for Tie2 and Ptie2 western blots of GC1. Figures 5 and S7 represent the quantification of 3 replicates shown here. Ladder markers sizes shown on left. Tie2 expected molecular weight/size is 140 kDa. P-Tie2 expected molecular weight/size is 125 kDa. GAPDH expected molecular weight/size is 36 kDa. Actin expected molecular weight/size is 43 kDa. *NT: not treated; C: control; 1: 1 μM of regorafenib; 2: 2 μM of regorafenib; -: not irradiated; +: irradiated.*

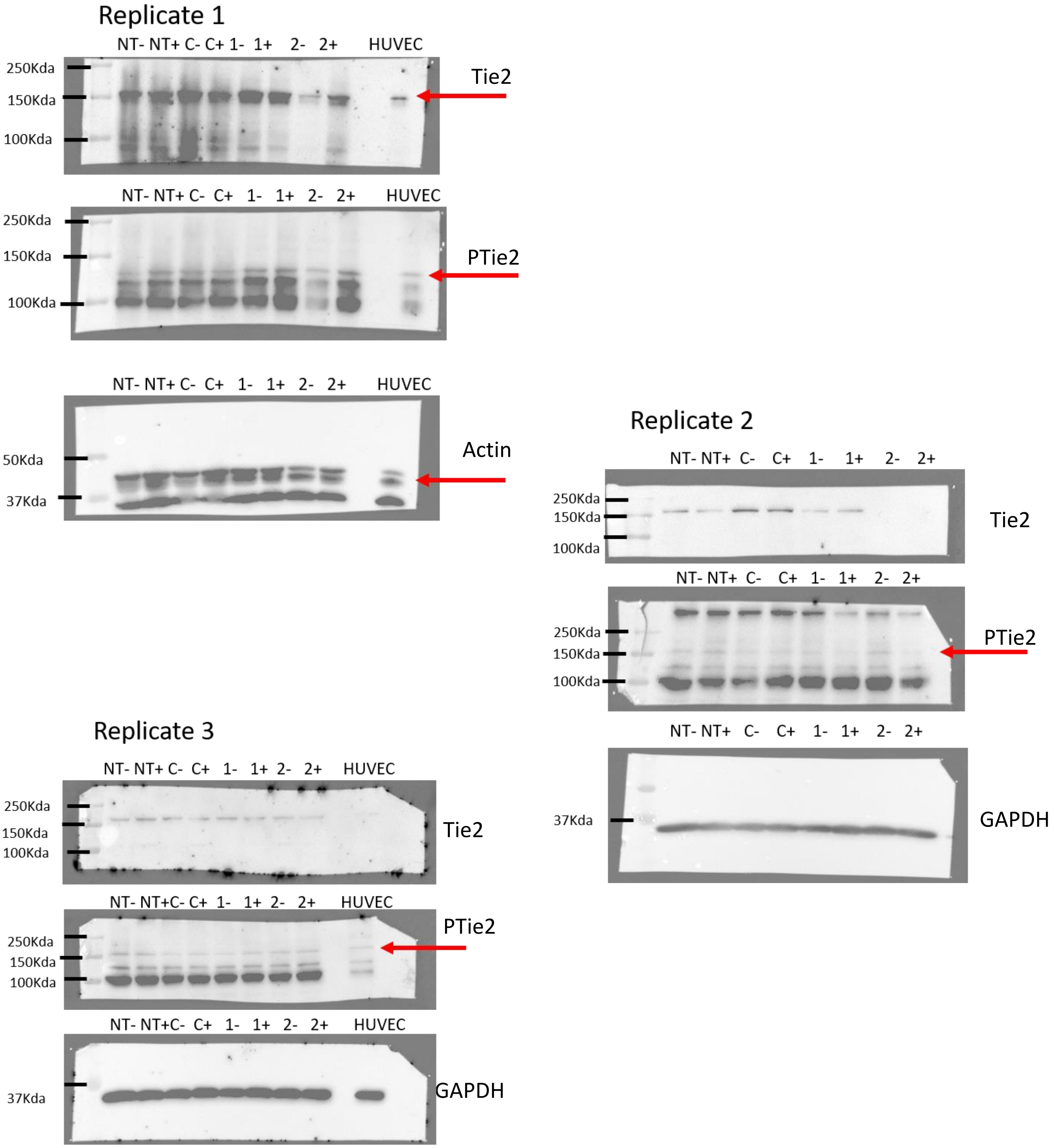

Supplementary Figure S13: Raw data for Tie2 and Ptie2 western blots of GC2. Figures 5 and S7 represent the quantification of 3 replicates shown here. Ladder markers sizes shown on left. Tie2 expected molecular weight/size is 140 kDa. P-Tie expected molecular weight/size is 125 kDa. GAPDH expected molecular weight/size is 36 kDa. . Actin expected molecular weight/size is 43 kDa. *NT: not treated; C: control; 1: 1 µM of regorafenib; 2: 2 µM of regorafenib; -: not irradiated; +: irradiated.*
